# MiRNA-26b inhibits proliferation by targeting PTGS2 in breast cancer

**DOI:** 10.1186/1475-2867-13-7

**Published:** 2013-02-01

**Authors:** Jia Li, Xiangjie Kong, Junfeng Zhang, Qifeng Luo, Xiaoyu Li, Lin Fang

**Affiliations:** 1 Department of Breast and Thyroid, Shanghai Tenth People’s Hospital, Shanghai, 200072, China

**Keywords:** MiR-26b, Proliferation, PTGS2, Breast cancer

## Abstract

**Background:**

MicroRNAs (miRNAs) are small, non-coding RNAs (20–24 nucleotides) that post-transcriptionally modulate gene expression by negatively regulating the stability or translational efficiency of their target mRNAs. The aim of this study was to investigate the expression pattern of microRNA-26b (miR-26b) in human breast cancer, and its potential role in disease pathogenesis.

**Methods:**

Quantitative reverse transcription-polymerase chain reaction (qRT-PCR) was performed to determine the expression level of miR-26b in 38 breast cancer specimens and adjacent normal breast tissues. MTT assays were conducted to explore the impact of miR-26b overexpression on the proliferation of human MDA-MB-231 breast cancer cells. Luciferase reporter assays were employed to validate regulation of a putative target of miR-26b. The effect of modulating miR-26b on endogenous levels of this target were subsequently confirmed via qRT-PCR and Western blot.

**Results:**

MiR-26b expression was relatively decreased in breast cancer specimens compared with adjacent normal tissues (*P*<0.01). Overexpression of miR-26b suppressed MDA-MB-231 cell growth. Luciferase assays using a reporter carrying a putative miR-26b target site in the 3' untranslated region of *PTGS2* revealed that miR-26b directly targets *PTGS2*. Overexpression of miR-26b led to downregulation of PTGS2 at the mRNA and protein level, as assessed by qRT-PCR and Western blot. Targeted knockdown of PTGS2 by siRNA significantly inhibited the proliferation of MDA-MB-231 breast cancer cells.

**Conclusions:**

MiR-26b may act as a tumor suppressor in breast cancer. The overexpression of miR-26b inhibits cellular growth by targeting *PTGS2*, suggesting its use as a potential therapeutic target for breast cancer.

## Introduction

MicroRNAs are a class of small, non-coding RNAs, which are capable of regulating gene expression at the post-transcriptional level. Mechanistically, miRNAs function by binding to the 3^′^ untranslated regions (UTRs) of target mRNAs, causing translation to be blocked and/or mRNA degradation [1]. MicroRNAs play diverse roles in tumorigenesis and in the progression of breast cancer, and may act as oncogenes, tumor suppressors and modulators of tumor proliferation, invasion, apoptosis and therapy resistance [2-6]. An increasing body of evidence indicates that miR-26b is downregulated in hepatocellular carcinoma [7], nasopharyngeal carcinoma [8], primary squamous cell lung carcinoma [9], squamous cell carcinoma of the tongue [10] and in breast cancer [11]. Furthermore, overexpression of miR-26b induces apoptosis in MCF-7 breast cancer cells by targeting SLC7A11 [11]. However, to date, the role of miR-26b in breast cancer tumorigenesis is incompletely understood.

*Prostaglandin-endoperoxide synthase-2* (*PTGS2*) encodes the COX-2 enzyme, which catalyzes the conversion of arachidonic acid to prostaglandins (PGs) and other eicosanoids. PTGS2 expression, which is undetectable in most normal tissues, is induced in response to hypoxia, inflammatory cytokines, tumor promoters, growth factors and other stressors [12,13]. PTGS2 is involved in carcinogenesis, immune response suppression, inhibition of apoptosis, angiogenesis and tumor cell invasion and metastasis. Recent studies have indicated that PTGS2 genetic variation is associated with breast cancer susceptibility [14,15]. Furthermore, overexpression of PTGS2 in patients with breast cancer is associated with a worse prognosis [16]. Several studies have demonstrated the involvement of miRNAs in the regulation of PTGS2. MiR-199a and miR-101a are implicated in PTGS2 regulation during embryo implantation [17]. Furthermore, miR-26b has been shown to directly silence PTGS2 and regulate PTGS2 expression in desferrioxamine (DFOM)-treated carcinoma of nasopharyngeal epithelial (CNE) cells [8].

In this study, we report that miR-26b expression is significantly decreased in human breast cancer, and its overexpression inhibits the proliferation of MDA-MB-231 cells by targeting PTGS2. These results indicate that miR-26b functions as a tumor suppressor, whose dysregulation may be involved in the initiation and development of human breast cancer.

## Results

### Expression of miR-26b is decreased in human breast cancer

**Figure 1:**
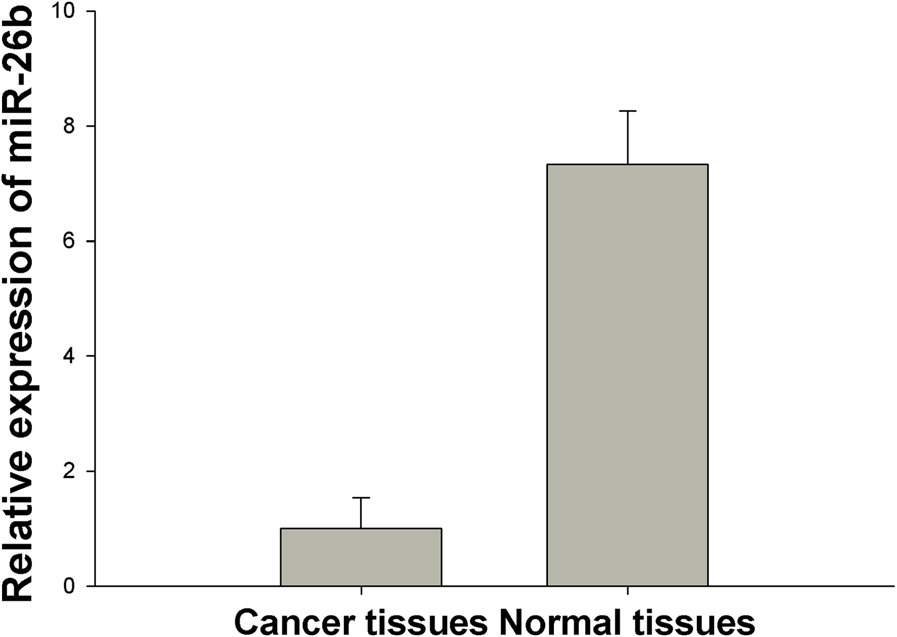
miR-26b is relatively downregulated in breast cancer.

To investigate the involvement of miR-26b in breast cancer development, we analyzed levels of miR-26b in 38 invasive ductal breast cancer tissues and associated normal adjacent tissues (NATs) by quantitative reverse transcription-polymerase chain reaction (qRT-PCR). MiR-26b expression was significantly decreased in breast cancer tissues compared with NATs (7.3 fold, *P*<0.01) (Figure 1).

### Suppression of breast cancer proliferation by miR-26b

**Figure 2:**
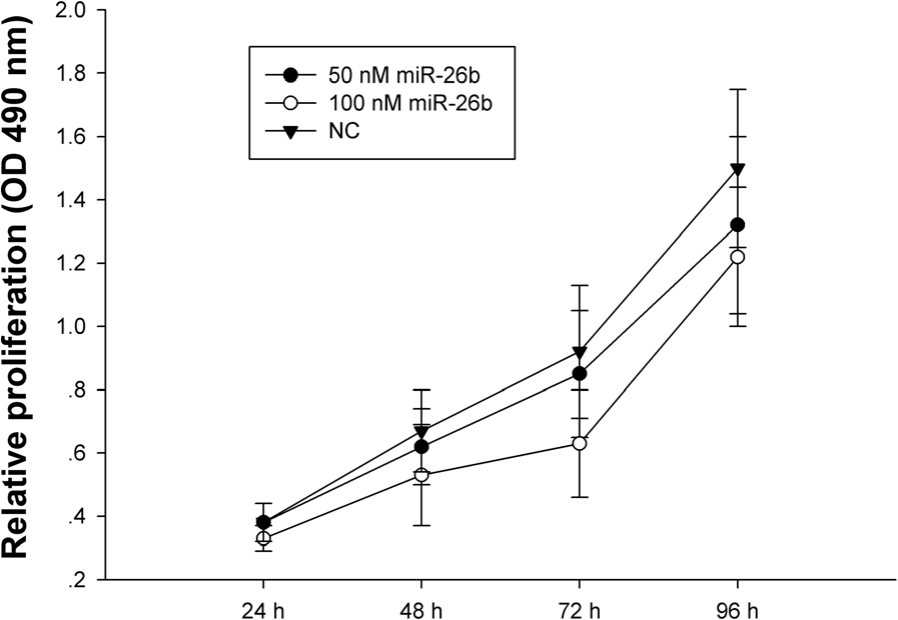
miR-26b inhibits the proliferation of breast cancer cells.

To investigate the effect of miR-26b on breast cancer cell proliferation, miR-26b mimics were transfected into the human breast cancer cell line, MDA-MB-231 and proliferation was assessed by MTT assay. As shown in Figure 2, cellular proliferation gradually declined following transfection with miR-26b, in a concentration-dependent manner. Treatment of cells with 50 nM miR-26b led to a decrease in MDA-MB-231 cell growth at 72 h (7%) and 96 h (18%) (*P*<0.05) compared with the negative control. This inhibitory effect was significantly enhanced following transfection with 100 nM miR-26b at 48 h (14%), 72 h (29%) and 96 h (28%) (*P*<0.05) compared with the negative control. Taken together, these results demonstrate that miR-26b inhibits the proliferation of MDA-MB-231 breast cancer cells.

### MiR-26b regulates PTGS2 expression in breast cancer cells

**Figure 3:**
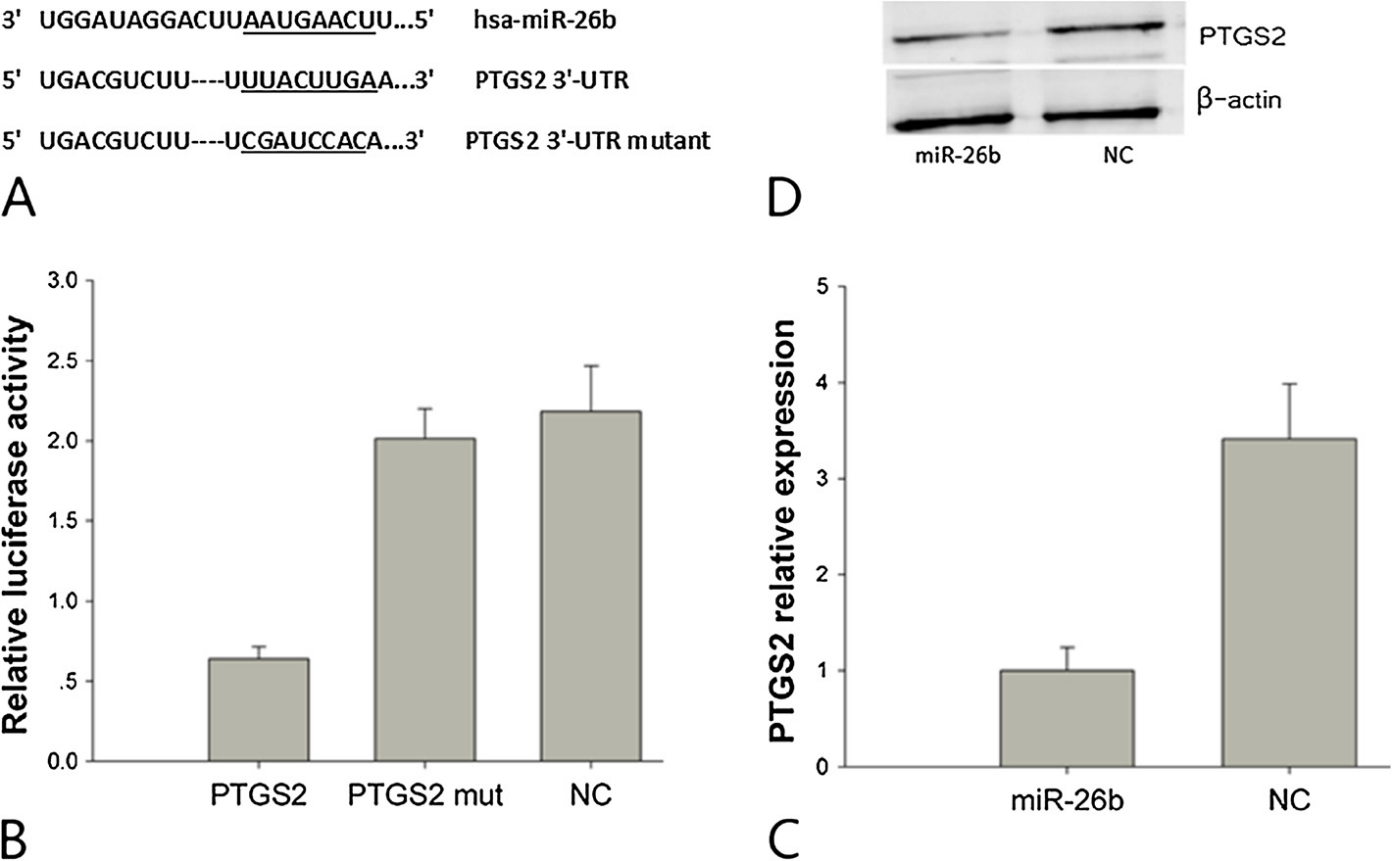
PTGS2 is a direct target of miR-26b in breast cancer.

To investigate the downstream targets of miR-26b that may play a role in mediating this growth suppressive effect, we searched for putative targets using the miRanda database. We identified a binding site for miR-26b in the 3^′^-UTR of *PTGS2* mRNA. To validate miR-26b binding to this predicted site, we cloned the 3^′^-UTR of *PTGS2* containing the putative miR-26b binding site into a luciferase reporter construct, in addition to a mutated *PTGS2* 3^′^-UTR (Figure 3A). Luciferase activity was significantly decreased following co-transfection of psiCHECK-2/PTGS2 3^′^-UTR with miR-26b, compared with the miR-negative control (miR-NC) (Figure 3B). Furthermore, luciferase activity was also decreased following co-transfection of psiCHECK-2/PTGS2 3^′^-UTR mutant and miR-26b (Figure 3B). These results indicate that miR-26b specifically binds to the 3^′^-UTR of *PTGS2*. The effect of miR-26b transfection on endogenous *PTGS2* mRNA and protein expression was subsequently evaluated in MDA-MB-231 cells by qRT-PCR and Western blot. As shown in Figure 3C and 3D, the expression of *PTGS2* mRNA and protein was decreased in MDA-MB-231 cells transfected with 100 nM miR-26b mimics compared with the control. These results suggest that miR-26b directly targets PTGS2 in breast cancer cells.

### MiR-26b inhibits the proliferation of breast cancer cells via regulation of PTGS2

**Figure 4:**
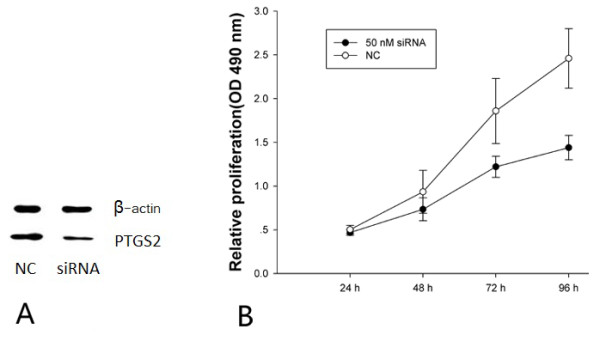
knockdown of PTGS2 significantly supresses the proliferation of breast cancer cells.

Since overexpression of miR-26b suppressed the proliferation of MDA-MB-231 breast cancer cells, and given that PTGS2 is a direct target of miR-26b, we hypothesized that the inhibitory effect of miR-26b on breast cancer cell viability might be achieved via targeting PTGS2. To investigate this, we assessed the effect of targeted knockdown of PTGS2 on MDA-MB-231 cell growth by MTT assay. Treatment of cells with 50 nmol/L PTGS2 siRNA markedly suppressed cell viability by 21%, 34% and 41% at 48 h, 72 h and 96 h, respectively, compared with control siRNA (*P*<0.01) (Figure 4). This suggests that PTGS2 promotes the proliferation of breast cancer cells *in vitro*. These results demonstrate that downregulation of PTGS2 expression by miR-26b contributes, at least in part, to the suppression of the growth of breast cancer cells.

## Discussion

The discovery of the first miRNA, lin-4, in *Caenorhabditis elegans* initiated a new era of miRNA biology. Since then, thousands of miRNAs have been identified and annotated. Furthermore, an increasing body of evidence indicates that miRNAs are differentially expressed between normal and tumor tissues, suggesting that dysregulation of miRNA expression is a key factor underlying tumorigenesis [18-22]. In this study, we performed qRT-PCR to investigate the expression pattern of miR-26b in primary human breast cancer. MiR-26b expression was significantly downregulated in breast cancer specimens compared with normal tissue. Similar findings have been reported in several other cancer types, including hepatocellular carcinoma [7], nasopharyngeal carcinoma [8], primary squamous cell lung carcinoma [9], squamous cell carcinoma of tongue [10] and glioma [23]. Consistent with this study, Xiao-Xiao Liu *et al.* reported that miR-26b expression is downregulated in MCF7, HCC1937, MDA-MB-231, MDA-MB-468, MDA-MB-453, BT-549 and BT-474 breast cancer cell lines compared with CCD-1095Sk normal breast skin cells [11]. These results indicate that miR-26b is downregulated in human breast cancer specimens and cell lines.

It is well established that regulation of gene expression by miRNAs plays a role in the development, differentiation, proliferation, apoptosis, invasion and metastasis of a variety of cancers. In our study, transfection of miR-26b mimics into MDA-MB-231 cells led to a significant decrease in cellular proliferation, indicating that miR-26b represses the growth of breast cancer cells. Previous studies demonstrated that overexpression of miR-26b in DFOM-treated CNE cells inhibited proliferation via degradation of *PTGS2* mRNA and suppression of PTGS2 protein translation [8]. Gain- and loss-of-function studies showed that miR-26b and its host genes CTDSP1/2/L cooperate to block G1/S-phase progression by activating pRb protein in hepatocellular carcinoma [24]. MiR-26b is also involved in processes governing apoptosis in breast cancer. Previous studies reported that miR-26b mimics triggered apoptosis of human breast cancer MCF7 cells, and SLC7A11 was identified as a direct target of miR-26b [11]. In glioma, low levels of miR-26b were inversely correlated with tumor grade. Ectopic expression of miR-26b inhibited the proliferation, migration and invasion of human glioma cells, possibly via regulation of its downstream target, EphA2 [23]. Taken together, these studies indicate that dysregulated expression of miR-26b may affect multiple cancers.

Computational algorithms revealed that the 3^′^-UTR of *PTGS2* contains a binding site for miR-26b. To confirm targeting of PTGS2 by miR-26b, we integrated a fragment of the *PTGS2* 3^′^-UTR containing the target sequence, or a fragment whose target site was mutated, into a luciferase reporter vector. Luciferase activity was significantly repressed in cells transfected with the construct harboring the miR-26b target sequence compared with the mutated control vector. Both *PTGS2* mRNA and protein levels decreased after transfection of MDA-MB-231 cells with miR-26b, as shown in Figure 3C and 3D. These data indicate that miR-26b directly interacts with *PTGS2* mRNA and represses PTGS2 protein expression. Furthermore, silencing PTGS2 expression by siRNA led to inhibition of cellular proliferation. In conclusion, these findings support the hypothesis that decreased expression of PTGS2 by miR-26b accounts for the suppression of cellular proliferation in breast cancer.

## Conclusions

Taken together, we demonstrate that miR-26b is downregulated in breast cancer specimens compared with normal tissue. MiR-26b directly downregulates PTGS2 and inhibits breast cancer cell proliferation. These data indicate that miR-26b may serve as a tumor suppressor gene involved in breast cancer pathogenesis.

## Materials and methods

### Specimens

In this study, 38 paired breast cancer and normal specimens were collected from the Department of Breast and Thyroid Surgery of Shanghai Tenth People’s Hospital, Shanghai, China. All samples were confirmed as invasive, ductal breast cancer by trained pathologists. No patients received chemotherapy or radiotherapy prior to surgery.

### Cell culture

The MDA-MB-231 breast cancer cell line was purchased from the Chinese Science Institute. Cells were maintained in Dulbecco’s Modified Eagle’s Medium (DMEM) (Gibco, USA) supplemented with 10% Fetal Bovine Serum (FBS) (Gibco), penicillin (100 units/ml) and streptomycin (100 μg/ml) (Enpromise, China). Cells were incubated at 37°C in a humidified chamber supplemented with 5% CO_2_.

### qRT-PCR

For detection of *miR-26b* expression, primer design and qRT-PCR was performed as previously described [25]. MiRNA was isolated using the miRcute miRNA isolation kit according to the manufacturer’s instructions (Tiangen, China). *MiR-26b* was amplified using the following primers: 5^′^-GTCGTATCCAGTGCAGGGTCCGAGGTATTCGCACTGGATACGACGAGCCA-3^′^ (stem-loop primer), 5^′^-CGCCCTGTTCTCCATTACTT-3^′^ (sense) and 5^′^-CCAGTGCAGGGTCCGAGGT-3^′^ (antisense). Amplification of control U6 small nuclear RNA was performed using the following primers: 5^′^-GTCCTATCCAGTGCAGGGTCCGAGGTGCACTGGATACGACAAAATATGGAAC-3^′^ (stem-loop primer), 5^′^-TGCGGGTGCTCGCTTCGCAGC-3^′^ (sense) and 5^′^-CCAGTGCAGGGTCCGAGGT-3^′^ (antisense). cDNA was generated by reverse transcription using the PrimeScript™ RT-PCR kit in accordance with manufacturer’s instructions (Takara, Japan). Real-time PCR was performed on a 7900HT fast RT-PCR instrument (Applied Biosystems, Singapore). PCR parameters for miRNA quantification were as follows: 2 min at 95°C, followed by 40 cycles of 30 s at 95°C and 45 s at 60°C.

For quantification of *PTGS2* mRNA expression, total RNA was isolated using TRIzol (Invitrogen, USA), and cDNA was generated by reverse transcription using the PrimeScript RT-PCR kit in accordance with the manufacturer’s instructions (Takara). Real-time PCR was performed on a 7900HT fast RT-PCR instrument using SYBR-Green and the following primers: *PTGS2,* 5^′^-CCTGTGCCTGATGATTGC-3^′^ (sense), 5^′^-CTGATGCGTGAAGTGCTG-3^′^ (antisense); *β-actin,* 5^′^-AGCAGCATCGCCCCAAAGTT-3^′^ (sense) and 5^′^-GGGCACGAAGGCTCATCATT-3^′^ (antisense). The PCR parameters for relative quantification were as follows: 2 min at 95°C, followed by 40 cycles of 15 s at 95°C and 30 s at 60°C. Each sample was tested in triplicate. The relative expression was calculated using the relative quantification equation (RQ) = 2-ΔΔCT [26].

### Western blot analysis

Protein samples were separated by 12% SDS-polyacrylamide gel (SDS-PAGE) and transferred onto PVDF membranes (Beyotime, China). Immune complexes were formed by incubation of membranes with primary antibody (BioVision, USA) overnight at 4°C. Blots were washed and incubated for 1 h with HRP-conjugated anti-rabbit secondary antibody. Immunoreactive protein bands were detected using an Odyssey Scanning system.

### MTT assay

MDA-MB-231 cells (5000/well) were plated in 96-well plates (BD Biosciences, USA) and incubated at 37°C overnight. The next day, sub-confluent (50-60%) cells were transfected with miR-26b mimics (50 nmol/L and 100 nmol/L) or PTGS2 siRNA (50 nmol/L) (Genepharma, China) using Lipofectamine 2000 (Invitrogen, USA), in accordance with the manufacturer’s instructions. MiR-NC and siRNA control were used as negative controls. DMEM medium was replaced with DMEM supplemented with 10% FBS 5 h post-transfection with miR-26b mimics or PTGS2 siRNA. Cell proliferation was assessed at 24, 48, 72 and 96 h, using the MTT proliferation assay kit in accordance with the manufacturer’s instructions (Sigma, USA). All experiments were performed in biological triplicate.

### Luciferase assay

The 3^′^ untranslated region (3^′^ UTR) of *PTGS2* containing the predicted miR-26b binding site was amplified by PCR in a total volume of 50 μl using the Primer star kit (Takara) in accordance with the manufacturer’s instructions. The primers used were 5^′^-TAGGCGATCGCTCGAGCTGTTGCGGAGAAAGGAGTC-3^′^ (sense); 5^′^-AATTCCCGGGCTCGAGTAGTTACTTCTAATGCATCATGG-3^′^ (antisense). The mutant constructs were generated by mutation. Fragments were subcloned into the Xho I site in the 3^′^-UTR of Renilla luciferase of the psiCHECK-2 reporter vector. psiCHECK-2/PTGS2 3^′^-UTR or psiCHECK-2/PTGS-2 3^′^-UTR mutant reporter plasmids (200 ng) were co-transfected with miR-26b mimics or miR-NC (100 nM) into MDA-MB-231 cells (60% confluence) using Lipofectamine 2000 (Invitrogen), in accordance with the manufacturer’s instructions. After 48 h, cells were lysed and reporter activity was assessed using the Dual-luciferase reporter assay system (Promega, USA) in accordance with the manufacturer’s protocols. Renilla luciferase activity was normalized to firefly luciferase activity.

### Statistical analysis

Data are presented as the mean ± standard deviation from at least three independent experiments. The two-tailed *t*-test was used to draw a comparison between groups. The null hypothesis was rejected at the 0.05 level.

## Abbreviations

miRNAs: MicroRNAs;RT-PCR: Quantitative Reverse Transcription-Polymerase Chain Reaction; PTGS2: Prostaglandin-endoperoxide synthase-2; FBS: Fetal bovine serum; DMEM: Dulbecco’s modified Eagle’s medium; SDS-PAGE: SDS-polyacrylamide gel; NATs: Normal adjacent tissues.

## Competing interest

The authors declare no conflict of interest.

## Authors’ contributions

LF designed and directed the study, XK and JZ conducted western blotting, QL and XL performed qRT-PCR, JL performed luciferase reporter assays and drafted the manuscript. All authors read and approved the final manuscript.
