# Correction: MiRNA-26b inhibits proliferation by targeting PTGS2 in breast cancer

**DOI:** 10.1186/1475-2867-13-17

**Published:** 2013-02-21

**Authors:** Jia Li, Xiangjie Kong, Junfeng Zhang, Qifeng Luo, Xiaoyu Li, Lin Fang

**Affiliations:** 1 Department of Breast and Thyroid, Shanghai Tenth People’s Hospital, Shanghai, 200072, China

## Correction

After publication of this article [1], the authors noted an error in Figure http://1. The labelling of normal tissues and cancer tissues was inverted. Please see Figure 1 for the corrected version. The authors apologise for any inconvenience this has caused.

**Figure 1:**
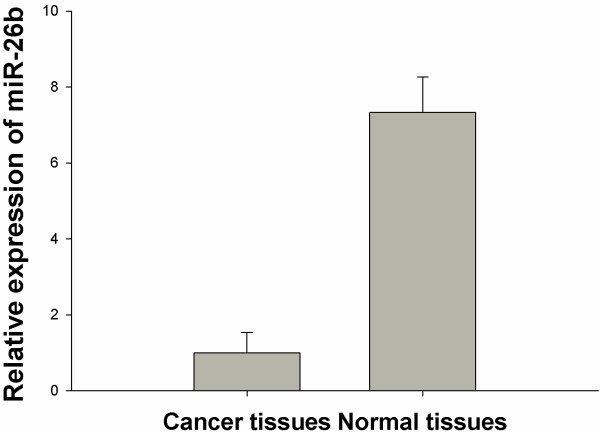
miR-26b is relatively downregulated in breast cancer.
